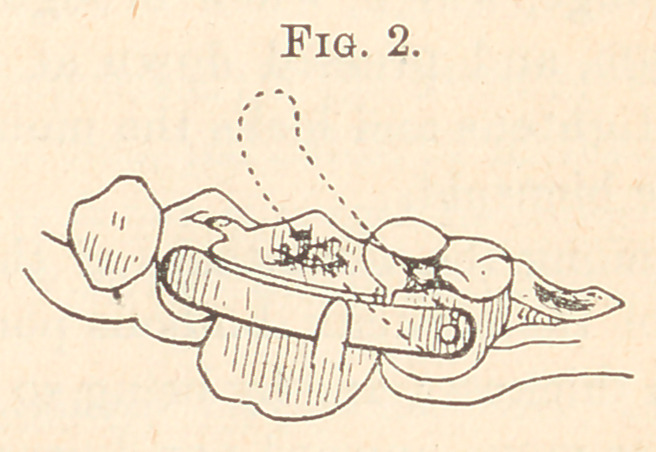# Latch-Lock, Lever-Clasp, Plates, and Bridges

**Published:** 1894-07

**Authors:** Wm. S. Davenport

**Affiliations:** Paris, France


### LATCH-LOCK, LEVER-CLASP, PLATES, AND BRIDGES.[1]

BY WM. S. DAVENPORT, D.D.S., PARIS, FRANCE.

FIGS. 1 and 2 illustrate two pieces of removable bridges, which are retained in position by means of *hinged levers* as *latches.*

FIG. 1.

FIG. 2.

Number one has been in use for eight months and number two for five months, during which time they have given perfect satisfaction. It is needless to say, in the making of the same every detail must be accurately carried out. In each case an impression was taken of the molar to which the piece was to be attached. This was accurately secured by the use of plaster of Paris, which was broken from the tooth and the pieces reunited.

---

[1] Read before the American Dental Club, of Paris, April 7, 1894.

Fusible metal was poured into the impression, giving an absolute fac-simile of the tooth. A platinum band was burnished around this die, so as to conform to the convexity of the crown of the tooth.

A small *platinum wire* was then bent around the band in place, at its largest circumference, and its ends twisted together; its position was marked, the wire was slipped off, the platinum band sprung off the metal tooth.

The wire was then replaced on the band, and the united band and wire, after being invested in sand and plaster, was covered to a desired thickness with Williams's clasp-metal, used as a solder, the whole making a tough and springy clasp.

The platinum wire not only preserves the form of the band, but acts as a guide for the even distribution of the clasp-metal over the surface of the platinum band; the thickness of the band being regulated by the size of the wire. This band was then cut open at the point near where the hinged lever was to be placed. A wire post was soldered to one end, to which the hinge was subsequently attached. An impression was taken on that side of the mouth with the band in position on the natural tooth, the porcelain facing, necessary clasps, and the lock-post were arranged on the model and waxed in position.

The piece was then removed, invested, and soldered as in ordinary bridge-work. The lever, consisting of a strong piece of clasp-metal, was riveted to the pin on the free end of the clasp, making the hinge, which, when brought behind the lock-post, acting as a fulcrum, and pressed down at the labial side of the bicuspid, not only tightens and locks the molar band, but clasps the piece firmly to the bicuspid.

Among the advantages of this method of clasping is the utilization of the corneux, bulbous portion of the teeth, where the enamel is the thickest; and by being so locked to the corneux surface it can neither move upward nor downward, but is easily removed by raising the lever.

## Figures and Tables

**Fig. 1. f1:**
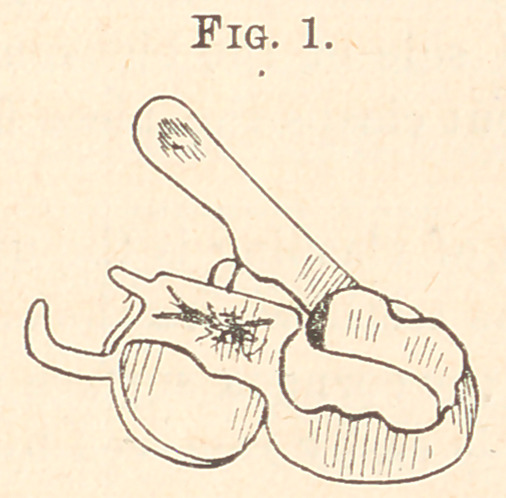


**Fig. 2. f2:**